# Topiramate-induced acute onset myopia: a case report

**DOI:** 10.1186/1756-0500-7-665

**Published:** 2014-09-21

**Authors:** Arjuna Medagama, Tissa Senaratne, Jayasuriya Mudiyanselage Ruwanthi Panchali Bandara, Rajitha Asanga Abeysekera, Imbulpitiya Vidanalage Buddhini Imbulpitiya

**Affiliations:** Department of Medicine, Faculty of Medicine, University of Peradeniya, Peradeniya, Sri lanka; General Hospital, Kandy, Sri lanka; Teaching hospital, Peradeniya, Sri Lanka

**Keywords:** Topiramate, Acute myopia, Ciliochoroidal effusion syndrome

## Abstract

**Background:**

Topiramate is a drug which emerged from its anticonvulsant properties and now over the years is used for a wider range of indications, including migraine prophylaxis. We described a very rare case of topiramate induced acute onset myopia during use for migraine. It is the first reported case of its kind from Sri Lanka with only a handful of reported cases in world literature.

**Case presentation:**

A 35-year-old Sri Lankan female presented with long standing history of intermittent headache with recent worsening. A diagnosis of migraine was made and due to poor response to other medication was initiated on topiramate. Two weeks later patient developed visual impairment which was finally attributed to topiramate. Following discontinuation of the drug, within 3 days the symptoms started to improve with full recovery in 10 days.

**Conclusion:**

All clinicians should be aware of the potential ocular side effects of topiramate. Although relatively rare, prompt recognition is key to appropriate management.

## Background

Topiramate (TPM) emerged from its anticonvulsant properties and now is recommended as a first line medication in some seizure disorders. Over the years its use has significantly increased and is used for a wide spectrum of other indications including migraine prophylaxis.

Topiramate is a sulfa-derivative monosaccharide with several mechanisms of action, mainly including blockage of voltage-gated sodium channels, hyperpolarization and enhancement of postsynaptic gamma-aminobutyric acid receptor activity [1]. It is a drug with numerous side effects, in particular ophthalmologic side effects [2]. Due to increased use it is prudent that the clinician is fully aware of these potential side effects. We describe here a rare case of topiramate induced acute onset myopia. The author feels it is the first reported case of its kind from Sri Lanka despite having reported cases in world literature [3].

## Case presentation

A 35-year-old Sri Lankan female with long standing intermittent headaches presented with recent worsening of headache. Clinical examination performed by the physician revealed normal neurological examination and vision with a visual acuity of 6/6 without any correction. A provisional diagnosis of migraine has been made on a previous occasion and she had been commenced on multiple medications over a period of time including propranolol and flunarazine. This had helped initially, but not in the long term.

The patient was reassured and asked to continue flunarazine. She presented one month later with no relief from her headaches. Examination was unremarkable. Flunarazine was discontinued and she was commenced on topiramate 25 mg at night.

2 weeks later she complained of acute onset blurring of vision. There were no other neurological symptoms. An ophthalmological consultation revealed no significant visual finding except for a bilateral refractive error of – 0.5 diopters. Her visual symptoms persisted to the next day, but did not worsen. Suspecting topiramate induced myopia the drug was discontinued and the patient was referred back to the ophthalmologist who noted a visual acuity of 6/6, normal intraocular pressure and a worsening of refractive error to −3.5 diopters bilaterally. On the 3rd day following discontinuation of topiramate her visual symptoms started to improve and at the end of 10 days her vision was back to normal with no evidence of a refractive error (0 diopters).

A diagnosis of topiramate induced acute myopia was made. An ophthalmologist review 2 weeks later was normal.

## Discussion

This patient had no other previous medical illness or visual disorders prior to the commencement of topiramate. The symptom onset was very acute in nature following commencement of topiramate and improved spontaneously on discontinuation of the drug.

The exact mechanism by which TPM and other sulfa derivatives initially triggers myopia is not completely understood. However, to date, a number of mechanisms have been suggested as possible triggers.

One of the earliest hypotheses was proposed by Sen *et al*. [4]. They suggested that the entry of TPM into the lens alters its osmotic status, causing it to swell and, consequently, resulting in angle closure glaucoma (ACG) and myopia. Another hypothesis by Ikeda *et al.* [5] in 2002 led to the origin of the clinical entity “Ciliochoroidal effusion syndrome” which can be defined as a spectrum of clinical manifestations ranging from transient topiramate induced myopia to severe bilateral ACG . These complications were attributed to ciliochoroidal effusion and swelling of the ciliary body, which can potentially result in anterior rotation of the ciliary processes, causing narrowing of the ciliary sulcus and forward displacement of the iris and lens.

Another theory is that Ciliochoroidal effusion syndrome is due to an idiosyncratic reaction. This complication occurring in a small proportion and some studies showing no correlation between TPM dosage with the level of intra ocular pressure or myopia supports this.

Patients with topiramate induced myopia often complain of sudden blurred vision. The symptoms are documented to occur within the first 2 weeks after drug initiation. As the mean plasma elimination half life of the drug is about 21 hours, rapid visual recovery usually occurs although in some cases it can take several weeks. In the management of topiramate induced myopia, the most important measure is to discontinue TPM and seek urgent ophthalmology opinion. In the long term an alternative agent should be used.

## Conclusion

Ocular examination before starting topiramate cannot identify eyes at risk. Patients commencing topiramate should therefore be advised to immediately report any symptoms of eye pain or blurred vision especially in the first few weeks of treatment.

All clinicians involved in the management of the patient including the physician, neurologist and ophthalmologists need to be aware of the potential ocular side effects of topiramate. Although relatively rare, prompt recognition is key so appropriate management can be instituted and visual outcomes minimized.

## Consent

Written informed consent was obtained from the patient for publication of this Case Report and any accompanying images. A copy of the written consent is available for review by the Editor-in-Chief of this journal.

## Abbreviations

TPM: Topiramate
ACG: Angle closure glaucoma.

## References

[1] Perucca E (1997). A pharmacological and clinical review on topiramate, a new antiepileptic drug. Pharmacol Res.

[2] Panday VA, Rhee DJ (2007). Review of sulfonamide-induced acute myopia and acute bilateral angle- closure glaucoma. Compr Ophthalmol Update.

[3] Bhattacharyya KB, Basu S (2005). Acute myopia induced by topiramate: report of a case and review of the literature. Neurol India.

[4] Sen HA, O’Halloran HS, Lee WB (2001). Case reports and small case series: topiramate-induced acute myopia and retinal striae. Arch Ophthalmol.

[5] Ikeda N, Ikeda T, Nagata M, Mimura O (2002). Ciliochoroidal effusion syndrome induced by sulfa derivatives. Arch Ophthalmol.

